# Ongoing Activity in Temporally Coherent Networks Predicts Intra-Subject Fluctuation of Response Time to Sporadic Executive Control Demands

**DOI:** 10.1371/journal.pone.0099166

**Published:** 2014-06-05

**Authors:** Takayuki Nozawa, Motoaki Sugiura, Ryoichi Yokoyama, Mizuki Ihara, Yuka Kotozaki, Carlos Makoto Miyauchi, Akitake Kanno, Ryuta Kawashima

**Affiliations:** 1 Smart Ageing International Research Center, Institute of Development, Aging and Cancer, Tohoku University, Sendai, Japan; 2 Department of Functional Brain Imaging, Institute of Development, Aging and Cancer, Tohoku University, Sendai, Japan; 3 Japan Society for the Promotion of Science, Tokyo, Japan; University Of Cambridge, United Kingdom

## Abstract

Can ongoing fMRI BOLD signals predict fluctuations in swiftness of a person’s response to sporadic cognitive demands? This is an important issue because it clarifies whether intrinsic brain dynamics, for which spatio-temporal patterns are expressed as temporally coherent networks (TCNs), have effects not only on sensory or motor processes, but also on cognitive processes. Predictivity has been affirmed, although to a limited extent. Expecting a predictive effect on executive performance for a wider range of TCNs constituting the cingulo-opercular, fronto-parietal, and default mode networks, we conducted an fMRI study using a version of the color–word Stroop task that was specifically designed to put a higher load on executive control, with the aim of making its fluctuations more detectable. We explored the relationships between the fluctuations in ongoing pre-trial activity in TCNs and the task response time (RT). The results revealed the existence of TCNs in which fluctuations in activity several seconds before the onset of the trial predicted RT fluctuations for the subsequent trial. These TCNs were distributed in the cingulo-opercular and fronto-parietal networks, as well as in perceptual and motor networks. Our results suggest that intrinsic brain dynamics in these networks constitute “cognitive readiness,” which plays an active role especially in situations where information for anticipatory attention control is unavailable. Fluctuations in these networks lead to fluctuations in executive control performance.

## Introduction

The concept of brain being an intrinsically driven system [1] has recently gained attention and attracted research efforts. Intrinsic neural activity, which manifests as fluctuations in the functional magnetic resonance imaging (fMRI) blood-oxygen level dependent (BOLD) signals, exposes the temporally coherent networks (TCNs) or intrinsic connectivity networks that correspond to various known functions [2]–[6]. These TCNs have been consistently observed during rest and task performance, and their inter-individual differences correlate with differences in various cognitive and psychological functions [7]–[10]. Furthermore, as an evidence of functional and behavioral relevance of intrinsic neural activity at an individual level, fluctuations in BOLD signals have been shown to correlate with intra-subject fluctuations in the performance of perceptual and motor tasks [11]. Indeed, in some task-relevant regions or TCNs, ongoing BOLD signals immediately before the onset of a trial can predict the outcome of subsequent somatosensory [12], auditory [13], visual [14], [15], and motor [16], [17] processes.

The impact of these intrinsic brain dynamics on the outcomes of higher-order cognitive processes that require executive control remains unknown. More specifically, it remains to be determined whether ongoing BOLD signals in TCNs predict intra-subject performance fluctuations in response to sporadic high-executive-control demands. A general expectation is that high ongoing pre-trial activity in an executive-control-related network would function as a “readiness” activity for executive control and facilitate successful performance. Previous studies [18]–[21] have indicated the involvement of two interacting but functionally separable network groups in executive control: the cingulo-opercular network (CON) and the fronto-parietal network (FPN). The CON has been implicated in the stable maintenance of task set and goals. The FPN has been observed to respond more rapidly to control demands and to initiate and adjust control on a trial-to-trial basis. Therefore, ongoing activity of the TCNs in these two groups is expected to have different effects on subsequent executive control. In contrast, higher pre-trial activity in TCNs that are deactivated during the task could lead to deteriorated performance. The default mode network (DMN), the activity of which increases when individuals are not focused on the external world [22]–[24], is representative of these task-negative networks.

The purpose of the current study was to explore the predictive effects of pre-task activity in these executive-control-related TCNs on subsequent executive control performance. In contrast to previous fMRI studies addressing similar predictive effects, our study aimed to determine the functional significance of ongoing activity on executive control with higher sensitivity by focusing on the following aspects: (1) minimizing inter-trial dependency and (2) raising the demand on executive control.

With regard to inter-trial dependency, several studies [25]–[27] have shown that prestimulus activity in several regions of the CON, FPN, or DMN can predict fluctuations in the performance of simple tasks requiring executive control. However, these studies used similar task settings in consecutive trials with short trial-to-trial onset asynchrony, which would have made the onset of the next trial predictable and the outcome of one trial influencing the next. Therefore, it remains unclear whether BOLD fluctuations that were predictive of executive control performance originate from ongoing intrinsic brain dynamics, or are dominated by anticipatory attention, performance monitoring, maladaptation, and attention reorienting processes that occur in a combined sequence of trials [25]–[27].

To reduce inter-trial dependency, a recent study [28] used a color–word Stroop task of sparsely timed trials with variable and unpredictable intervals (20−40 s). The results showed that ongoing activity in the dorsal anterior cingulate cortex (dACC) and dorsolateral prefrontal cortex (dlPFC) along with ventral visual areas could be predictive of subsequent response speeds for the task. However, the predictive effect was observed only in some subjects who showed a behavioral Stroop interference effect, using a regions-of-interest (ROIs) analysis that was limited to several regions specified to be task-relevant. A possible reason for this limited effect was the simplicity of the task set, which suggests the need for higher demand on executive control. Task set is a specific cognitive state that a person enters in order to conduct a task at hand, and executive control is assumed to play key roles in implementing, configuring, and maintaining a task set [18], [29]. In the standard Stroop task used in the previous study [28], the required task set was very simple: “ignore the word.” Therefore, it is possible that the response speed for this task was not sensitive enough to detect the contribution of ongoing activity in various networks to the fluctuations in executive control. Another possible issue related to inter-trial dependency is the inter-trial interaction caused by congruent and incongruent conditions. Experiencing congruent trials in a Stroop task affects the task set and the conflict effect in subsequent incongruent trials [30], [31]. Even in a sparse-event-related design, the inclusion of congruent trials may add another source of variance in executive control performance, making it more difficult to detect the effects of ongoing activity.

Addressing these points, we conducted an fMRI study using a version of the color–word Stroop task that was designed to put a higher load on executive control in order to make its fluctuations more detectable. Incongruent color–word stimuli were used for all trials to avoid the possible inter-trial interactions due to inclusion of the congruent condition. To enhance the independence between trials, the task trials were sparsely timed, and they had long, variable, and unpredictable intervals. TCNs were extracted from BOLD data obtained during the performance of the task and during rest. We investigated the relationships between the activity of the TCNs around the onset of the task trial and the trial-by-trial variation in the task response time (RT). Performing the Stroop task requires selective attention, inhibition of prepotent responses, maintenance of task set, cognitive flexibility, and processing speed. RT has been widely used to evaluate these aspects of executive control [25], [28], [30]–[33]. Specifically, we defined TCNs that predict cognitive performance as those in which fluctuations in prestimulus signals significantly explained RT variance of the subsequent trials.

Our primary hypothesis was that high ongoing pre-trial activity in an executive-control-related TCN would work as a “preparatory” activity and facilitate successful performance (short RTs). Particularly, TCNs constituting the CON were expected to show high and persistent performance predictivity because of the increased load on executive control imposed by the task and the increased importance of task-set maintenance, as well as the network’s major role in conflict resolution and response inhibition [34]. The TCNs included in the FPN are involved in top-down visuospatial attention [35] and bottom-up attention reorienting [36]. Due to the task-driven nature of these processes in an uncued setting, the performance predictive effects of these TCNs could be transient. On the other hand, the performance predictivity of the DMN TCNs was expected to be the inverse of the executive control TCNs; their ongoing activity could reflect disengagement from the external environment [22]–[24], and would lead to slower responses. In addition, we also expected performance predictivity in the perceptual and motor-related TCNs that should be controlled by the executive TCNs while conducting the task.

## Materials and Methods

### Participants

This study was approved by the Ethics Committee of Tohoku University Graduate School of Medicine, and has been conducted according to the Declaration of Helsinki. Written informed consent was obtained from all participants. Forty-eight healthy, right-handed Japanese university students (20 females and 28 males; age: 19−24 years, mean ± SD: 20.3±1.4) participated in this study. Handedness was evaluated using the Edinburgh Handedness Inventory [37]. The subjects had normal or corrected-to-normal vision, and reported no history of neurological or psychiatric conditions.

### Task and Procedure

**Figure 1:**
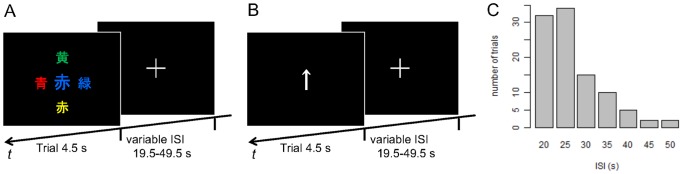
Experimental paradigm. (A) The Stroop task for fMRI sessions. In each trial, subjects were required to indicate the position of a surrounding word that names the font color of the central word. All the words used were color–word incongruent. (B) Control task outside the scanner. Subjects were required to simply replicate the directions indicated by the arrows. (C) Distribution of inter-stimulus intervals (ISIs) across trials for both tasks.

In fMRI sessions, we used a modified version of the color–word Stroop task. In our task, a stimulus for each trial consisted of a color-naming Kanji (Chinese character) word at the center of the screen, and four smaller color-naming Kanji words surrounding the central one, all naming and colored by either blue (“
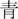
”), green (“
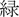
”), red (“
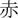
”), or yellow (“
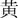
”), on a black background (Figure 1A). All the words used were color–word incongruent. Based on previous studies [38], [39], we expected that this task, consisting of a number of conflicting components and requiring the subject to switch attention to different aspects of the stimuli (first color and then word; see the explanation below), would place an even higher demand on executive control compared with the usual color–word Stroop task setting. Stimuli were projected onto a translucent screen that was viewed by the subjects through a mirror attached to the head coil. The center word subtended a 0.69° visual angle, and the surrounding words 0.53°, and the diameter from the center of the screen to the center of the surrounding words formed a 0.88° visual angle. Subjects were required to find, from the four surrounding words, the one that named the font color of the center word, and indicate its direction (up, down, left, or right) by operating a MR-compatible joystick device (RTC Joystick, Resonance Technology Inc.) with their right hand. They were instructed to minimize errors and respond as quickly as possible, and also to fix their eyes on the center of the screen, rather than search the surrounding words in a sequential manner. In each trial, the stimulus was presented for 4.5 s and subjects were asked to respond during this period.

To ensure that the task trials were as independent as possible from each other, and to exclude the exertion of anticipatory attention control as much as possible, the presentation of trials was made sporadic and unpredictable by long and random inter-stimulus intervals (ISIs) in the range of 19.5−49.5 s (mean: 26.8 s). Figure 1C shows the distribution of ISIs, which was basically the same as that used in a study addressing the effect of prestimulus intrinsic activity on the outcome of visual perception [14]. These settings resulted in trial-to-trial onset asynchrony varying from 24 s to 54 s (mean: 31.3 s), a much more sparse and unpredictable condition compared with previous studies, which used mean trial-to-trial onset asynchrony of approximately 6 s [25], [26], [40], [41]. During the ISIs, subjects were instructed to keep their eyes on the fixation cross at the center of the screen. Each stimulus presentation was synchronized to one of the trigger signals from the scanner, which informed the start of volume acquisition. The stimulus presentation and response recording were controlled by in-house software.

Each individual was subjected to four fMRI task sessions, each lasting approximately 13 min and containing 25 trials. Before the experiment, subjects performed 150 self-paced practice trials of the same Stroop task on a computer outside the MRI scanner. Between the fMRI task sessions, as a control task, subjects also conducted four sessions of a simple response task on a computer outside the scanner. In this task, subjects were required to answer the direction of presented arrow stimuli (up, down, left, or right; length 1.91° visual angle) using a joystick device (Figure 1B). ISI settings, duration of sessions, and the number of trials were the same as for the fMRI task. Due to technical reasons, log data of the simple response task for two subjects was incomplete. Therefore, they were excluded from the analyses related to the simple response task. Between all the sessions, subjects had self-paced breaks of 5 min or longer.

### Analyses on Behavioral Data

To check whether Stroop task performance was essentially independent between trials, we tested the auto-correlation of RTs between successive trials. Linear auto-correlation of lag 1 was calculated for each session of each subject, converted to a *z*-score using Fisher’s *r*-to-*z* transformation, averaged over sessions, and its significance over subjects was then tested using a two-tailed one sample *t*-test. To confirm that the pre-trial ISIs did not significantly affect RTs of the subsequent trials, we tested the correlations between RTs and the preceding ISIs in the Stroop task sessions. Pearson’s correlation coefficient was calculated for each subject over sessions, subjected to Fisher’s *r*-to-*z* transformation, and its significance over subjects was tested using a two-tailed one sample *t*-test.

### Image Acquisition

MRI data were collected using a 3-T Philips Intera Achieva scanner equipped with an 8-channel head coil. The fMRI BOLD signal was measured with a first field echo-echo planar imaging (FFE-EPI) sequence (64×64 matrix, TR = 1500 ms, TE = 30 ms, flip angle = 75°, FOV = 192 mm, slice thickness = 4.5 mm, gap = 0.5 mm, 25 trans-axial slices per volume). For the task sessions, 520 functional volumes were acquired in each run. Resting-state BOLD activity before and after task sessions was also recorded for each subject. For each of the two resting sessions, 240 functional volumes were acquired. Scanning parameters were the same as those used in task sessions. During the resting-state scanning, subjects were instructed to remain immobile with their eyes closed, keep as motionless as possible, stay awake, and not think about anything in particular. Head motion was restricted using a pillow, a band, and foam cushions.

### Preprocessing of Functional Images

All EPI images were (1) realigned to the first image in the time series to correct for head movement; (2) slice-time corrected with the first slice of each volume as a reference slice; (3) normalized to the Montreal Neurological Institute (MNI) reference space using an EPI template and resliced to a cubic voxel size of 3 mm^3^; and (4) smoothed by a Gaussian kernel with 8 mm FWHM, using SPM8 (Wellcome Department of Cognitive Neurology, London, UK, http://www.fil.ion.ucl.ac.uk/spm/). In the preprocessing phase of the independent component analysis (ICA) explained below, the data were further intensity-normalized (i.e., converted to percent-signal-change units by dividing the time series of each voxel by its average intensity), to improve the accuracy and reliability of ICA [42].

### TCN Extraction

To extract TCNs from fMRI data, we used spatial ICA, which has been shown to extract consistent and reproducible TCNs [3], [43]–[46]. Unlike seed-based approaches, ICA does not require predefined ROIs. This is appropriate for the current study because we were open to the possibility that even TCNs that are not overtly involved in task execution could exert effects on the trial-by-trial fluctuations in task performance.

We extracted TCNs from brain activity that occurred during the Stroop task sessions, as well as during the resting sessions. Group ICA was performed on fMRI data from all 48 subjects, including data from the six (four task and two resting) sessions for each individual, using the GIFT toolbox (http://mialab.mrn.org/software/gift/). ICA model order (number of components) was set to 53, based on the mean number of independent components estimated in each session for each subject by minimum description length (MDL) criteria modified to account for spatial correlation [47]. To reduce the computational load for ICA, a two-step data reduction by principal component analysis (PCA) was applied to the preprocessed fMRI data [44], [48]: the first step reduced the data of each subject and each session into *T*_1_ = 83 principal components (PCs), which were then spatially concatenated, and the second PCA reduced it to C = 53 PCs. Then, Infomax ICA [49] was applied 100 times using Icasso and the centrotypes of the independent component (IC) clusters were extracted as stable estimates of aggregate ICs [50]. Icasso also gives a cluster quality index for *I_q_* each IC, which provides a measure of how stable the ICA decomposition was for the specified setting [50]. The above procedure was repeated with different values of the first step PCA order in the range of 53–106, and *T*_1_ = 83 was chosen as the setting that gives the most stable decomposition (i.e. the highest average *I_q_* value). Subject- and session-specific spatial maps and time courses were back-reconstructed using the GICA algorithm [48], with no additional scaling of the result. The time courses of back-reconstructed single subject-level TCNs from the task sessions were analyzed to identify performance predictive TCNs, as explained in the section below. We did not use the recently developed GICA3 algorithm [51], because it can be sensitive to PCA reduction components and its use for higher model order (>50) was not currently recommended by the developers of the GIFT toolbox.

The resulting group-level ICs were classified into TCNs or artifacts by inspecting their spatial maps and the power spectral characteristics of the time courses [42]. For spatial maps, we based our classification on (1) whether the peak voxels were located in the gray matter and (2) how similar their patterns were to known TCNs or to vascular, ventricular, motion, and susceptibility artifacts [6], [52]–[54]. For power spectra of ICs, we assessed two metrics, dynamic range (DR) and low to high power ratio (LH), for the IC time courses of the resting sessions. These metrics have been used previously to classify components extracted from resting-state fMRI data [42], [55]; both of these values were generally higher for TCNs than for artifacts. DR was defined as the difference between the maximum and minimum of the spectral power distribution for each time course. LH was defined as the ratio of the integral of power in the region of the spectrum below 0.02 Hz to the total [55]. Each metric was averaged for the two resting sessions, and for all subjects, to summarize the spectral characteristics of each IC.

### Functional Network Connectivity Among TCNs

To understand the functional roles of the TCNs through the temporal relationships between their ongoing activities, we additionally evaluated functional network connectivity (FNC) [56] during the resting and task sessions. The back-reconstructed time course of individual TCNs for each session and subject was submitted to linear detrending and Fourier band-pass filtering (0.0078–0.15 Hz), to reduce scanner-originated and physiological noise. For the task sessions, we also calculated FNC using the residual time courses from which the average trial-evoked response in each TCN was subtracted (see the next section). Functional connectivity between each pair of TCNs was derived by calculating zero-lag Pearson’s correlation coefficients between the preprocessed time courses for each session and subject. The correlation coefficients were subjected to Fisher’s *r*-to-*z* transformation and averaged separately for the two resting sessions and the four task sessions for each subject. The averages over subjects were calculated for each pair of TCNs, and the significance tested using a two-tailed one sample *t*-test.

### Statistical Analysis of Predictive Ongoing Activity

The same detrending and band-pass filtering as for the analysis of FNC were applied to the back-reconstructed time courses of individual TCNs for each task session and subject. Furthermore, average trial-evoked response in each TCN for each task session and subject was estimated by applying a finite impulse response (FIR) model to the processed time course. Twenty-five candlestick predictors were assigned for each scanning time point starting from the trial onset, and with an interval of TR = 1.5 s, modeling the period 36 s from the onset. This average response was regressed out from the processed TC, removing the expected after-effect of the previous trial (which was already small due to the long ISIs), and leaving a residual signal reflecting the trial-to-trial fluctuations in the activity of that TCN. The signal values at the 15 time points from −6 s to +15 s around the trial onsets were used as an explanatory variable in an analysis of covariance (ANCOVA) model explaining the variance of RT in the corresponding trials. This model also included other trial-associated variables as covariates: (i) session (1−4), (ii) trial (1−25), (iii) center word, (iv) word corresponding to the correct answer (font color of the center word), (v) font color corresponding to the correct answer, and (vi) position of the correct answer. The trial was treated as a continuous variable, whereas other factors were treated as categorical variables with three degrees of freedom. In summary, we estimated a general linear model with the residual signal variable and the covariates *x*s 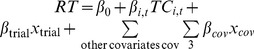 for each TCN (*i*) and each time point (*t*), for each subject. Error trials and time-out trials were excluded from the analysis.

The estimated values of the model coefficient *β_i,t_* were subjected to a random-effect statistical analysis, to explore TCNs for which ongoing pre-trial activity would predict RT fluctuations in the subsequent trials, as well as TCNs for which the trial-evoked response would be modulated in relation to RT fluctuations, at a population level. We performed a two-tailed one sample *t*-test on the group average of *β_i,t_*, for each TCN *i* and each time point *t*. Due to the exploratory nature of this study, the test results were considered significant at *p*<0.05 with control of the false discovery rate (FDR) for all the TCNs and time points.

The statistical tests were conducted using R statistical software [57].

## Results

### Behavioral Data

**Figure 2:**
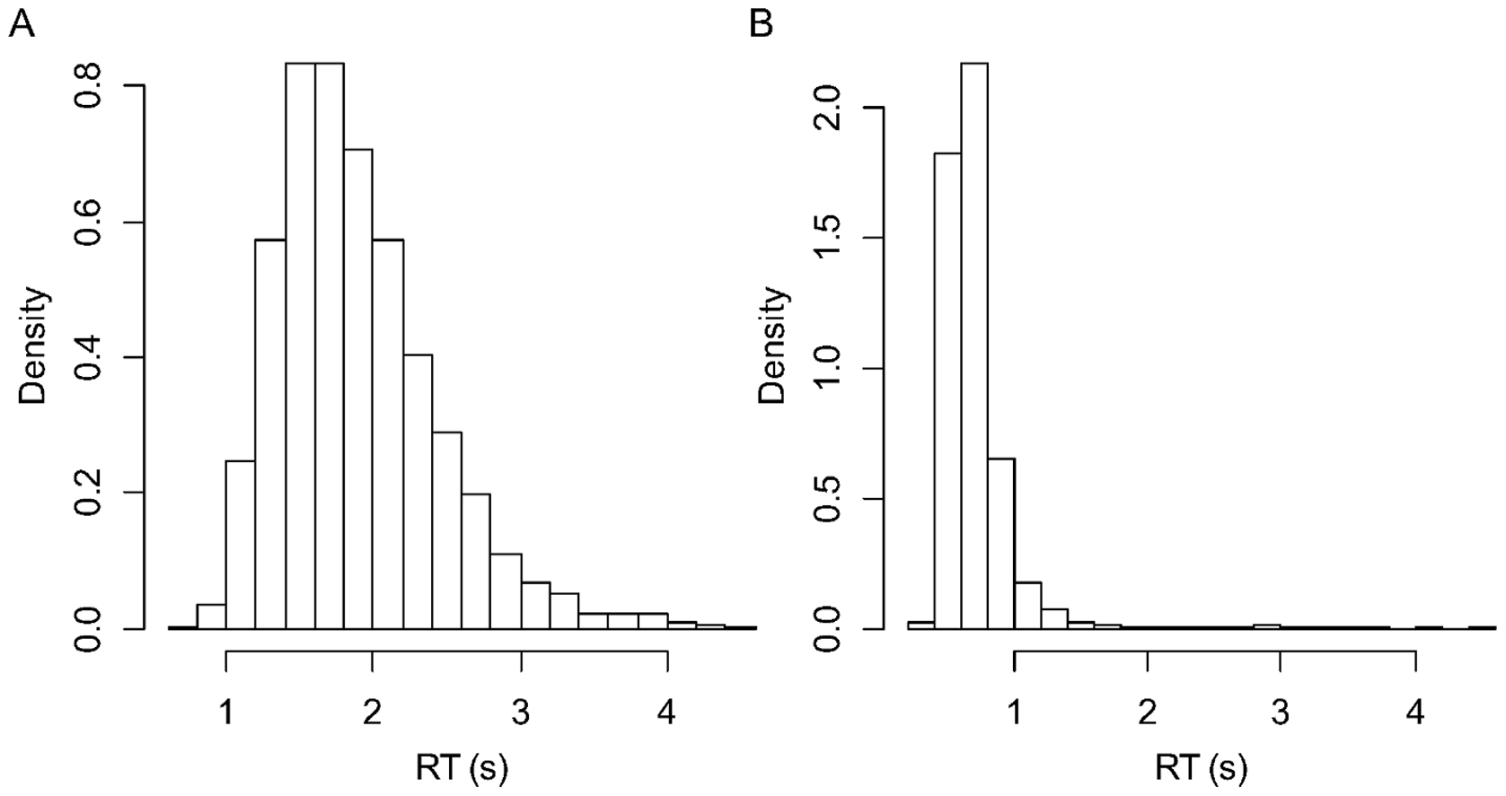
Distribution of response time (RT) for the Stroop task (A) and simple response task (B). Data of all subjects were concatenated.

Subjects performed both the Stroop task inside the scanner and the simple response task outside the scanner with high accuracy (mean accuracy was 0.976 and standard deviation (SD) was 0.026 for the Stroop task; mean accuracy was 0.983 and SD was 0.027 for the simple response task). There was no significant difference between the accuracy of the two tasks. For the Stroop task, mean RT was 1.91 s and SD was 0.47 s. For the simple response task, mean RT was 0.74 s and SD was 0.29 s. Both mean RT and SD were significantly higher in the Stroop task than in the simple response task (RT mean: *t*_paired_(45) = 28.96, *p*<2.2 ×10^−16^; RT SD: *t*_paired_(45) = 8.90, *p*<1.8 ×10^−11^). Since the distribution of RT data was skewed (Figure 2), we also tried analyses using log-transformed RT data, but found similar results. Therefore, we demonstrated the results using raw RT data.

There was no significant auto-correlation between RTs of successive Stroop trials (*t*(47) = −0.33, *p*>0.74). Moreover, there was no significant correlation between RTs and pre-trial ISIs (*t*(47) = −0.67, *p*>0.50).

### Extracted TCNs

Of the 53 independent components (ICs) obtained by group ICA, 18 ICs were identified as artifact components and the remaining 35 ICs were selected as TCNs (Table 1). Each IC had a cluster quality index greater than 0.8, indicating a highly stable ICA decomposition. Anatomical labels were given to the TCNs based on their spatial maps (Table 1). To facilitate the interpretation of results, the TCNs were classified into seven network groups: the CON, FPN, DMN, visual, auditory, sensorimotor, and subcortical networks, based on the overlap of their spatial maps with previously reported networks [18], [19], [42], [58]–[60] and their activation/deactivation to the task (Figure S1).

**Table 1:** All the independent components (ICs) extracted by the group ICA.

IC	*I_q_*	MNI peak			Spectral metrics		Label	Group
		x	y	z	DR	LH		
IC53	0.900	0	20	30	0.055	0.504	dACC	CON
IC38	0.933	42	18	−6	0.017	0.408	AI	CON
IC41	0.921	−54	16	−4	0.015	0.379	FO	CON
IC12	0.974	32	56	28	0.013	0.317	MFG/dlPFC	FPN
IC36	0.965	−54	14	36	0.013	0.330	l. MFG	FPN
IC50	0.866	56	20	34	0.013	0.342	r. MFG	FPN
IC39	0.955	−24	16	64	0.045	0.497	l. SFG/FEF	FPN
IC46	0.948	38	20	58	0.028	0.419	r. SFG/FEF	FPN
IC47	0.960	30	−2	70	0.036	0.363	SMA	FPN
IC28	0.955	−30	−64	64	0.014	0.334	SPL	FPN
IC09	0.968	0	−70	58	0.016	0.326	precuneus	FPN
IC30	0.965	−42	18	16	0.023	0.373	l. IFG Op	FPN
IC52	0.889	44	0	18	0.037	0.439	r. Rolandic Op	FPN
IC44	0.953	−56	−36	52	0.025	0.403	l. IPL	FPN
IC29	0.970	66	−28	30	0.048	0.480	r. SMG	FPN
IC02	0.979	0	50	−4	0.032	0.495	mPFC	DMN
IC49	0.899	0	−46	4	0.013	0.356	PCC	DMN
IC43	0.931	−12	−60	2	0.009	0.245	PCC+lingual	DMN
IC35	0.960	−62	−22	0	0.081	0.557	MTG	DMN
IC45	0.952	−54	−66	28	0.043	0.411	l. AG	DMN
IC31	0.971	62	−54	10	0.083	0.543	r. MTG	DMN
IC51	0.943	2	−84	40	0.015	0.395	cuneus	VIS
IC33	0.975	30	−70	44	0.018	0.212	SOG	VIS
IC08	0.973	4	−94	0	0.015	0.359	calcarine	VIS
IC03	0.976	26	−94	−14	0.006	0.252	lingual	VIS
IC07	0.974	−34	−64	−26	0.014	0.300	cerebellum	VIS
IC23	0.974	−46	−70	−8	0.108	0.609	l. FG	VIS
IC24	0.975	48	−64	−8	0.094	0.528	r. FG	VIS
IC21	0.967	−58	−22	16	0.084	0.560	l. SMG+STG	AUD
IC37	0.949	62	−16	8	0.021	0.398	r. STG	AUD
IC40	0.930	54	−4	52	0.007	0.269	l. SM	MOT
IC05	0.971	−36	−24	70	0.009	0.275	r. SM	MOT
IC04	0.971	48	−32	64	0.018	0.357	SM	MOT
IC13	0.966	62	−2	30	0.017	0.423	precentral	MOT
IC22	0.976	24	2	−14	0.013	0.332	BG+Amyg	SC
IC01	0.979	2	2	4	0.008	0.341	Artifact	
IC06	0.978	30	12	−30	0.016	0.325	Artifact	
IC10	0.974	14	38	58	0.019	0.301	Artifact	
IC11	0.976	0	−42	−34	0.019	0.423	Artifact	
IC14	0.963	−26	−36	76	0.006	0.148	Artifact	
IC15	0.968	46	8	−6	0.054	0.434	Artifact	
IC16	0.980	26	−40	18	0.069	0.505	Artifact	
IC17	0.964	−42	14	−2	0.013	0.289	Artifact	
IC18	0.975	−26	−44	14	0.013	0.232	Artifact	
IC19	0.972	−38	12	−16	0.009	0.280	Artifact	
IC20	0.974	4	−40	−2	0.009	0.233	Artifact	
IC25	0.974	−24	−40	28	0.024	0.344	Artifact	
IC26	0.970	2	−46	70	0.030	0.392	Artifact	
IC27	0.960	−32	−76	52	0.010	0.218	Artifact	
IC32	0.975	2	6	16	0.028	0.315	Artifact	
IC34	0.958	42	−66	54	0.023	0.340	Artifact	
IC42	0.959	32	−88	28	0.016	0.205	Artifact	
IC48	0.888	60	20	22	0.007	0.228	Artifact	

*I_q_*: cluster quality index; MNI peak: Montreal Neurological Institute (MNI) coordinates of the highest peak position; DR: dynamic range; LH: low to high power ratio.

ICs were classified into TCNs or artifacts, and TCNs were labeled with anatomical names based on their spatial maps. AG: angular gyrus; AI: anterior insula; Amyg: amygdala; dACC: dorsal anterior cingulate cortex; dlPFC: dorsolateral prefrontal cortex; FEF: frontal eye field; FO: frontal operculum; IFG: inferior frontal gyrus; IPL: inferior parietal lobule; MFG: middle frontal gyrus; mPFC: medial prefrontal cortex; MTG: middle temporal gyrus; PCC: posterior cingulate cortex; SFG: superior frontal gyrus; SMA: supplementary motor area; SMG: supramarginal gyrus; SOG: superior occipital gyrus; SPL: superior parietal lobule; Op: operculum;

TCNs were divided into the following groups based on their spatial organization and their activation/deactivation to the task: cingulo-opercular network (CON), fronto-parietal network (FPN), default mode network (DMN), visual (VIS), auditory (AUD), sensorimotor (MOT), and subcortical (SC) networks.

**Figure 3:**
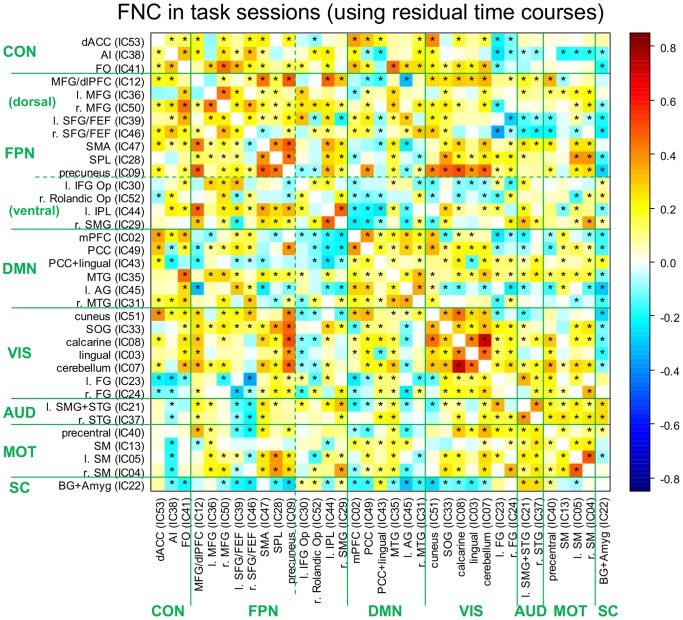
Functional network connectivity (FNC) between all temporally coherent networks (TCNs) for the ongoing activity time courses in the task sessions. The residual time course in each TCN of each subject was obtained by regressing out the average response from the back-reconstructed and preprocessed time course, leaving trial-to-trial fluctuation of the ongoing activity in the TCN. For each pair of TCNs, the correlation coefficient was calculated for each subject over sessions, subjected to Fisher’s *r*-to-*z* transformation, and averaged over subjects. The asterisks indicate significant connectivity over subjects (two-tailed one sample *t*-test, *p*<0.001 with control of the false discovery rate [FDR] for all TCN pairs). The solid lines indicate the division of TCNs into network groups. The FNC matrices (see also Figure S2) also support the division of the FPN into dorsal and ventral parts [64], [29], as indicated by the dashed lines. See the note below Table 1 for the abbreviations.

The FNC between all pairs of TCNs was evaluated by the correlations between the TCN time courses (Figure 3 and Figure S2). The connectivity pattern was largely consistent with the above classifications, with tighter connectivity between the TCNs within the same network group than between the different groups. On average, an anti-correlation tendency between the FPN and the DMN, as well as a positive correlation tendency between the CON and both the FPN and the DMN was observed in both the resting and task sessions. The results of one-sample *t*-tests (indicated by asterisks in Figure 3 and Figure S2) show that the patterns of connectivity between the TCNs were highly consistent over subjects.

### Predictive Ongoing Activity in TCNs

**Figure 4:**
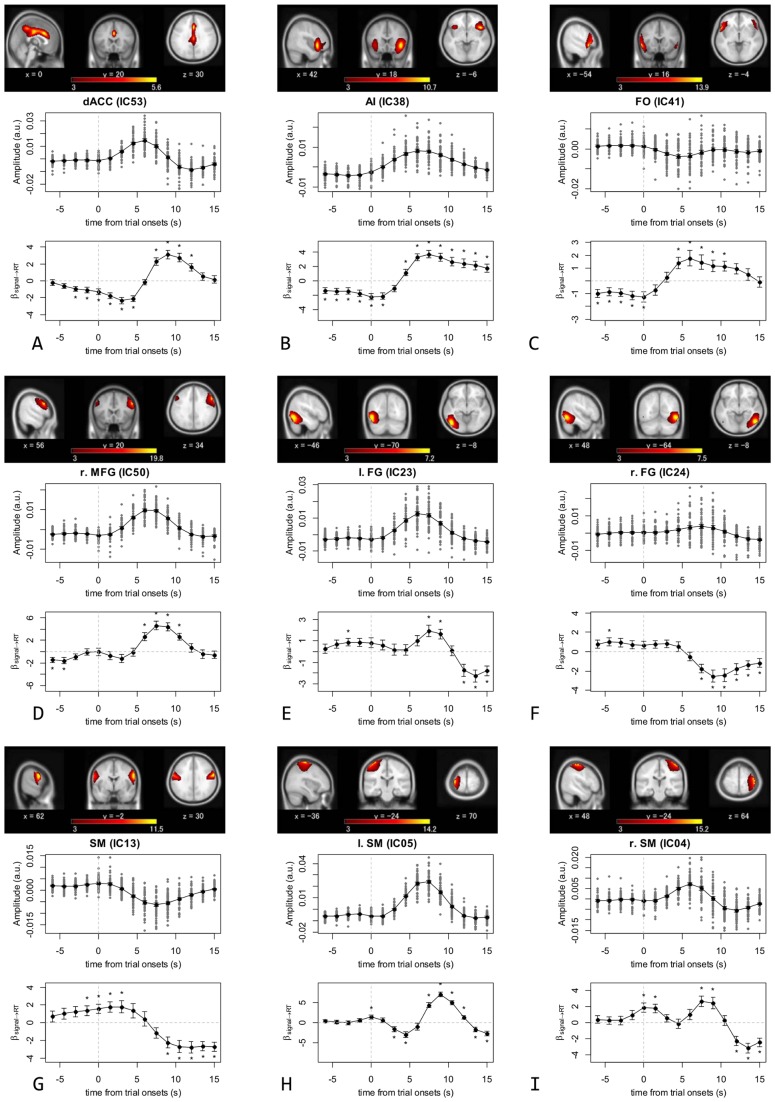
Description of the RT-predictive temporally coherent networks (TCNs). Spatial maps, BOLD activity around the trial onsets, and the relationship between their activity and the response time (RT) for the nine RT-predictive TCNs. (A) dorsal anterior cingulate cortex TCN (IC53); (B) anterior insula TCN (IC38); (C) frontal operculum TCN (IC41); (D) right middle frontal gyrus TCN (IC50); (E) left fusiform gyrus TCN (IC23); (F) right fusiform gyrus TCN (IC24); (G) sensorimotor TCN (IC13); (H) left sensorimotor TCN (IC05); (I) right sensorimotor TCN (IC04). For each TCN, a spatial map (converted to *z*-score and thresholded with *z* >3.0) shows its average distribution over all subjects and sessions, sectioned at the highest peak position [with its Montreal Neurological Institute (MNI) coordinates given], and superimposed on the MNI 152 standard space T1 template image. Dots in the BOLD activity represent pre-trial activity (time ≤ 0.0 s) and task response (time > 0.0 s) averaged over the trials in four task sessions for each subject, with the black line showing the grand average over all subjects. The time course for the relationship between activity and RT is shown as the group-averaged time course of estimated coefficients of an analysis of covariance (ANCOVA) model explaining the variance of RT. Error bars in the graphs show standard error of the mean (SEM) over subjects.

Analysis of the relationships between the activity of TCNs and RT fluctuations identified nine “RT-predictive TCNs”, for which the activity fluctuations before the trial onset (*t* ≤ 0.0 s) contributed significantly to explaining the RT fluctuations of the subsequent trials (Figure 4 and S1; Table 2). Three of these RT-predictive TCNs (IC53, 38, and 41) were classified into the CON, one (IC50) was in the FPN, two (IC23 and 24) were in the visual network, and three (IC13, 05, and 04) were in the sensorimotor network.

**Table 2:** Statistics for the response time (RT) predictivity of the nine temporally coherent networks (TCNs) at pre-trial time points (*t* = −6.0 to 0.0 s).

	−6.0 s		−4.5 s		−3.0 s		−1.5 s		0.0 s	
TCNs	*t*	*p*_FDR_	*t*	*p*_FDR_	*t*	*p*_FDR_	*t*	*p*_FDR_	*t*	*p*_FDR_
***Cingulo−opercular network (CON)***										
dACC (IC53)	−0.58	(0.725)	−2.01	(0.122)	−3.32	(0.007)	−3.39	(0.006)	−3.66	(0.003)
AI (IC38)	−3.20	(0.010)	−3.65	(0.003)	−3.33	(0.007)	−4.03	(0.001)	−5.06	(0.000)
FO (IC41)	−2.97	(0.016)	−2.73	(0.028)	−3.03	(0.014)	−3.42	(0.006)	−3.07	(0.014)
***Fronto−parietal network (FPN)***										
r. MFG (IC50)	−3.00	(0.015)	−3.02	(0.015)	−1.85	(0.158)	−0.09	(0.969)	−0.05	(0.975)
***Visual network (VIS)***										
l. FG (IC23)	0.66	(0.681)	1.94	(0.135)	2.46	(0.049)	2.42	(0.054)	1.84	(0.159)
r. FG (IC24)	1.79	(0.171)	2.46	(0.049)	2.17	(0.090)	1.76	(0.178)	1.68	(0.199)
***Sensorimotor network (MOT)***										
SM (IC13)	1.11	(0.438)	1.65	(0.206)	2.10	(0.103)	2.48	(0.047)	2.96	(0.016)
l. SM (IC05)	0.92	(0.534)	0.36	(0.856)	-0.07	(0.975)	1.40	(0.301)	3.10	(0.013)
r. SM (IC04)	0.78	(0.608)	0.60	(0.717)	0.53	(0.753)	1.69	(0.196)	3.25	(0.009)

Each cell shows the *t*-value (df = 47) of the two-tailed one sample *t*-test of the mean coefficient of RT-predictive ANCOVA model over subjects, with a braced false discovery rate (FDR)-corrected *p*-value.

The dACC TCN (IC53) centered on the dorsal part of the ACC and also extended to the posterior cingulate cortex (PCC; Figure 4A). It was activated by the task, and higher ongoing activity around *t* = −3.0 and 0.0 s predicted a quicker response, with the relationship strengthened further at *t* = +1.5 to +4.5 s. At approximately the peak of the activation (*t* = +6.0 s) the relationship was inverted, and a longer RT corresponded to larger post-peak activation.

The anterior insula (AI) TCN (IC38) centered around the insular cortex, with higher weight on the anterior part of the right insula (Figure 4B). The network was activated by the task, with higher ongoing activity as far back as 6.0 s before the trial onset persistently predicting a quicker response. The relationship between its activity and RT was inverted 4.5 s after the trial onset, but before peak activation, which occurred around 6.0 s from the onset. Thus, trials with longer RTs entailed higher or delayed task-evoked activation of this network.

The frontal operculum (FO) TCN (IC41) extended over the bilateral FO and inferior frontal gyrus (IFG) with higher weight on the left side, and a location exterior and anterior to the AI TCN (IC38) (Figure 4C). Although this network was slightly deactivated by the task, its higher ongoing activity from *t* = −6.0 to 0.0 s also predicted a quicker response, similar to the AI TCN. Its activity during the response period was positively correlated with RT, like that of the AI TCN.

The right middle frontal gyrus (r. MFG) TCN (IC50) extended mainly along the middle frontal gyrus (MFG), IFG, and the precentral gyrus, with larger involvement of the right hemisphere (Figure 4D). It showed clear activation by the task, and its response was positively modulated by RT, indicating that trials with a longer RT elicited a larger response in this network, whereas higher ongoing activity around *t* = −6.0 and −4.5 s predicted a quicker response.

The left and right fusiform gyrus (FG) TCNs (IC23 and 24) centered around the left and right FG, respectively (Figure 4E and F). The former was activated by the task, and its relatively higher ongoing activity at *t* = −3.0 s predicted a slower response. Its activity around the mean peak of activation was also positively correlated with RT. In contrast, the latter did not show a clear response to the task. Its relatively higher ongoing activity at *t* = −4.5 s predicted a slower response.

The sensorimotor (SM) TCN (IC13) covered the lateral and inferior parts of the sensorimotor areas bilaterally (Figure 4G). It was deactivated by the task, and relatively higher ongoing activity around *t* = −1.5 and 0.0 s predicted a slower response, with the relationship strengthened further at *t* = +1.5 and +3.0 s. At around *t* = +6.0 s the relationship was inverted, and a shorter RT was accompanied by faster recovery towards baseline activity.

The left and right SM TCNs (IC05 and 04) covered the left and right primary sensorimotor area, respectively (Figure 4H and I). They were activated by the task, and their relatively higher ongoing activity at *t* = 0.0 s predicted a slower response (longer RT). Their activity after the mean peak of activation was also positively correlated with RT, so that trials with longer RT coincided with higher or delayed task-evoked activation in these TCNs.

**Figure 5:**
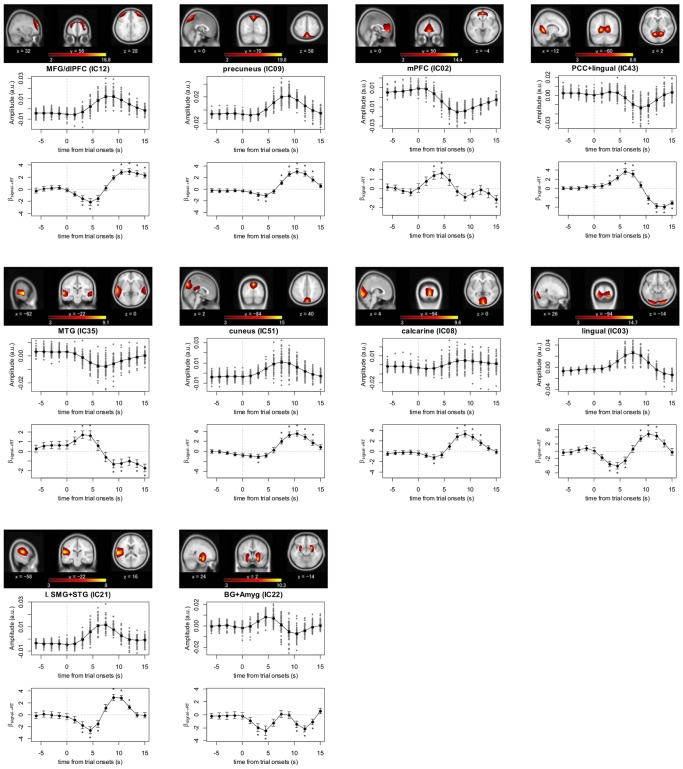
Description of temporally coherent networks (TCNs) that were partially RT-predictive. Ten TCNs for which the activity at 0.0< *t* ≤ 3.0 s from the trial onset significantly explained response time (RT) variability. The format for composite visualization of each TCN is the same as in Figure 4.

Considering the delay between the evoked hemodynamic signals and the actual electrophysiological neural activity, it has been suggested that ongoing activity states before the onset of trials may be reflected in the BOLD signals after the trial onset but well before the peak of the evoked hemodynamic response [16], [25]. Therefore, we also searched for TCNs for which the activity at 0.0 < *t* ≤ +3.0 s from the trial onset significantly explained RT variability. We identified ten such TCNs: two in the FPN [MFG/dlPFC (IC12) and precuneus (IC09) TCNs]; three in the DMN [medial prefrontal cortex (IC02), PCC+lingual gyrus (IC43), and middle temporal gyrus (IC35) TCNs]; three in the visual network [cuneus (IC51), calcaline (IC08), and lingual gyrus (IC03) TCNs]; one in the auditory network [left superior temporal and supramarginal gyri TCN (IC21)]; and one in the subcortical network [basal ganglia and amygdala TCN (IC22)] (Figure 5 and Figure S1). Task-evoked responses of the TCNs in the DMN were negative, while the other TCNs were “task-positive”, showing activation in response to the task. As a common feature, higher activity occurring at the early post-trial onset phase in the task-positive TCNs was accompanied by shorter RTs, while higher activity occurring after the activation peak was associated with longer RTs. This relationship was inverted for the TCNs in the DMN; their sustained activity in the early phase led to longer RTs, whereas quicker recovery of activity levels after deactivation tended to appear in trials with a shorter RT.

## Discussion

Our results revealed TCNs for which fluctuations in activity several seconds before the onset of trials predicted fluctuations in RT for the subsequent trials. These TCNs were prominent in the CON, and also distributed in the FPN, visual network, and sensorimotor network. Besides these, there were also TCNs in the FPN, DMN, and perceptual networks whose early post-trial onset signals explained RT fluctuations.

### Implications of Behavioral Results

In the behavioral results, the significantly larger mean and SD of RT in the Stroop task compared with the control task suggests that a greater part of RT variance was due to the fluctuations in cognitive aspects required by the task, especially executive control. Furthermore, the mean and SD of the RT was much larger than the values reported in previous studies that have addressed the effects of pre-trial activity on trial-by-trial performance variability [25], [26], [28], [40], [41]. This suggests that the task used in this study placed a higher load on executive control, and this in combination with differences in the task settings (sporadic and unpredictable vs. successive and predictable), made the fluctuations in executive control function more easily detectable.

Executive control tasks in a consecutive-trial setting have shown that the performance of one trial influences the subsequent trial, indicating the involvement of performance monitoring, maladaptation, and attention reorientation systems [25], [61]. In contrast, RT data in our study were virtually independent between successive trials. Moreover, if anticipatory attention control had affected executive control performance, we would have observed longer RTs in the trials with a longer pre-trial ISI because these were rarer and thus not easily anticipated. However, we did not observe a significant effect of pre-trial ISIs on RTs. These results support our expectation that the top-down adaptive systems did not play a major role in causing trial-by-trial RT fluctuations in our task setting.

### RT-predictive TCNs

#### Cingulo-opercular network

The RT-predictivity of the dACC TCN (Figure 4A) is consistent with previous studies [25], [28], which have also reported that relatively higher ACC activity around the trial onset predicts a shorter RT for visual attention and Stroop tasks. The present results, in which ongoing ACC activity several seconds before the onsets predicts the outcome of sparse and unpredictably presented Stroop task trials, may add further insight to discussions about the functional role of the ACC. The ACC is one of the most prominent regions activated during the color–word Stroop task as well as during similar executive control tasks [39], [62], [63]. There have been two predominant views concerning a major role for the ACC in performing these tasks: the maintenance of goal-oriented control and gating of irrelevant information, and the stimulus-driven detection and monitoring of conflicts and errors [33], [63], [64]. The current results support the former view, in contrast to some previous studies [25], [63], which have supported the latter.

The RT predictivity of the AI and FO TCNs (Figure 4B and C) is partially consistent with some previous results [25], but differs from others [28], and provides new information. Weissman et al. (2006) reported that higher activity in the right IFG around the trial onset (up to −1.25 to 0 s before the onset) predicts a shorter RT for a visual attention task [25]. With reference to this, we would like to stress two contrasting aspects to our results. Firstly, although higher pre-trial activity in both the AI and FO TCNs predicted a shorter RT for the Stroop task, the AI TCN was activated, whereas the FO TCN was not activated in response to the task. Secondly, the RT-predicting effects of ongoing activity were persistent and traced back further than previously reported, to −6.0 s before the trial onset. These two points strongly implicate the sustained, rather than task-driven nature of ongoing activity in these TCNs. In contrast, the study by Coste et al. (2011), which used a very similar sparse-event-related design but with the standard Stroop task, did not show RT predictivity in the regions corresponding to the AI and FO TCNs. The most likely explanation for the discrepancy would be the higher load on executive control, and thus the increased importance of task-set maintenance, for our task compared with the standard Stroop task, as we have hypothesized.

Consequently, the sustained RT predictivity of all constituent TCNs of the CON (Figure S1A) suggests that its role in stable task set maintenance [18], [19] is the key for the predictive effect of ongoing activity in this network on subsequent executive control performance. On the other hand, some studies that have focused on the relationships between the CON (or the salience network) and other networks, have argued against stable set maintenance as the network’s major function, rather emphasizing the role of the network (especially the right AI) in identifying salient stimuli and initiating transient control signals that lead to switching between the default mode and executive brain states [65]–[67]. Others have argued that the ACC plays a critical role in state maintenance, while the insula performs a major role in switching between states [68]. One way to reconcile such apparent inconsistencies may be by interpreting the role of the CON as implementing heightened arousal and awareness of the present [69], which can be either sustaining or transient depending on the dynamic reorganization of the CON’s interactions with other networks [21]. The exploration of such possibilities is beyond the scope of the present study, and will be the subject of future investigations.

#### Fronto-parietal network

The RT predictivity of the right MFG TCN (IC50; Figure 4D), along with the partial RT predictivity of the dorsolateral prefrontal (dlPFC) TCN (IC12; Figure 5 and Figure S1B) are generally compatible with previous studies [25], [28]. Weissman et al. (2006) reported that higher activity in the right MFG 1.25−2.5 s after the onset of trials predicted a shorter RT for the visual attention task [25]. Coste et al. (2011) reported that higher ongoing activity in the left dlPFC −1.5 s before the onset predicted a shorter RT on the Stroop task in a subject group that showed a stronger behavioral Stroop interference effect. On the other hand, the earlier time period for RT predictivity in the right MFG TCN (6.0 to 4.5 s before the onset) suggests that additional consideration is required to adequately interpret the specific role of this TCN. We hypothesized that due to the task-driven nature of the processes expected in the FPN [18]–[20] under the uncued task setting, the performance predictive effects in the FPN TCNs would be more transient.

The importance of attention switching in our task design needs to be taken into consideration. For a successful performance, our task required a quick shift of attention between different attributes of the stimuli: first the color of the center word (not prepotent and requires top-down attention), and then the semantic meaning of surrounding words (prepotent and salient). This would have increased the importance of rapid switching between top-down and bottom-up attention. The right MFG has been proposed as a hub node connecting dorsal and ventral fronto-parietal attention networks and is implicated in sustained attention, vigilance, and task set [4], [36]. The FNC pattern between the right MFG TCN and other FPN TCNs was also consistent with this (Figure 3 and Figure S2). We could speculate that RT-predictive ongoing activity in the right MFG TCN reflects mediation of the dorsal and ventral attention networks, leading to a state of preparedness for flexible attention switching. However, it is difficult to understand why RT predictivity in this TCN was not sustained until the trial onset.

#### Default mode network

We hypothesized that higher ongoing pre-trial activity in the DMN [22]–[24] reflects dissociation from the external task, and would lead to deteriorated performance (longer RTs). The results showed some TCNs in the DMN for which early post-trial onset signals showed significant predictivity on RT fluctuations in the expected direction (i.e. higher early activity led to a slower response), but their ongoing activity before the trial onset did not (Figure S1C). In previous studies, higher ongoing pre-trial activity in the DMN predicted errors [26], [27] but the effect on response time was not so significant [25], [28]. These observations suggest a possibility that the occurrence of errors is more sensitive to dissociation from external tasks than the RT. Another likely interpretation is that the dissociation from the task during the ISI was indeed “default” in this sparse and unpredictable task setting, and swift inhibition of these TCNs after the onset of the task was more important for achieving successful trials.

#### Visual network

The RT predictivity of the left fusiform (l. FG) TCN (Figure 4E) is consistent with the results of a previous study [28], which found that higher pre-trial activity in the visual word form area [70], a subregion of the left fusiform cortex contributing to the processing of visual word forms, led to slower response to the Stroop task. The result was interpreted in terms of the attentional bias toward the prepotent but inappropriate attribute (i.e. visual word form) over another appropriate attribute (i.e. color). Our results for the RT-predictive left and right FG TCNs (Figure 4E and F) can be interpreted in a similar manner. Temporarily heightened ongoing activity in these TCNs on the ventral visual pathway, possibly related to fluctuating top-down control, generated a bias toward processing of visual attributes such as word form or shape and interfered with the processing of color, which was also required at the first stage of our Stroop task.

On the other hand, in the visual as well as other networks, we also observed several task-positive TCNs for which higher activity at the early post-trial onset phase predicted shorter RTs (Figure 5; Figure S1D, E, and G). These TCNs also showed the “compensatory” response, i.e., the inverted relationship with RT in the later phase [25]. The higher early post-onset signal in these TCNs may have reflected either ongoing “preparatory” activity facilitating task-relevant functions such as visuospatial, language, and salience processing, or swifter recruitment of such functions in response to the task.

#### Sensorimotor network

The RT predictivity of the sensorimotor TCNs (Figure 4G, H and I; Figure S1F) is another unique result in this study. These TCNs were motor-related. More specifically, the two superior TCNs [left and right SM TCNs (IC05 and 04)] overlapped the representation areas of fingers on the sensorimotor somatotopic organization, and the bilateral and inferior SM TCN (IC13) overlapped the lip representation areas [71]. We refer to them as the right and left finger TCNs and the lip TCN, respectively. Note that the task required a response using the right hand, and the contralateral left finger TCN (Figure 4H) showed clearer activation than the ipsilateral right finger TCN (Figure 4I), whereas the task-irrelevant lip TCN showed deactivation (Figure 4G). Second, higher activity in these TCNs, specifically around the trial onsets, predicted longer RTs. Bilateral performance predictivity is consistent with previous studies [16], [17]. However, to the authors’ knowledge, the predictive effects of prestimulus activity in the sensorimotor TCNs on executive control performance have not yet been reported. For a task with high demands for conflict-resolution, higher ongoing activity in the finger TCNs could require additional control in order to suppress the urge for an immediate motor response, thereby leading to a slower response. Higher pre-trial activity of the lip TCN may have reflected distraction from the task, leading to a slower response.

### Limitations

The behavioral and imaging results have supported the use of the modified version of the Stroop task to achieve higher average cognitive load and larger variations in task performance, resulting in increased sensitivity for detecting RT-predictive ongoing activity in a wider range of TCNs. However, it also led to the involvement of additional aspects of executive control that are not included in the standard Stroop task: rapid switching of attention and visuospatial attention. As discussed above, the results of several RT-predictive TCNs could be interpreted in terms of attention switching and other executive control aspects such as task set maintenance, because their neural bases have not yet been clearly established. Additional studies will be needed to dissociate the contribution of ongoing activity in the RT-predictive TCNs to the fluctuations in those different aspects of executive control. Regarding visuospatial attention, we used visual stimuli with a small visual angle. In addition, the participants were asked to fixate on the center of the screen and not to search the surrounding words in a sequential manner. However, we could not objectively ensure their compliance with such directives (e.g. by using eye-tracking). Therefore, it is possible that a certain amount of the RT was devoted to the visual search process. On the other hand, we did not find RT predictivity in any of the TCNs that contain regions well-known for their involvement in visuospatial attention, such as the intraparietal sulcus and the frontal eye field [35], [72] (Figure S1B). This suggests that the fluctuations in visuospatial attention may not have had much impact on the observed RT fluctuations.

In this study, we used only incongruent color–word stimuli to avoid the possibility of an inter-trial interaction caused by the congruent condition [30], [31]. However, if a large enough number of trials were performed, these kinds of inter-trial interactions could be eliminated by discarding the first few trials that follow a congruent trial. Under those conditions, it would also be possible to address the effects of pre-trial activity in the TCNs on the Stroop congruency effect, by focusing on the incongruent trials just after a congruent trial.

Due to practical constraints, we administered the simple control task outside the scanner. However, fMRI data obtained during the control task may have been useful to help clarifying whether the observed RT-predictive effects in the sensorimotor TCN were due to fluctuations in local activity, or were caused by fluctuations in top-down control.

Due to the exploratory nature of this study and the small effect size that was anticipated in order to be comparable with previous results [25], [26], [28], we preferred to control the false discovery rate (FDR) for all the selected TCNs and time points. This strategy provides higher sensitivity but lower reproducibility in comparison to family-wise error rate (FWER) correction methods, such as the Bonferroni correction. Still, our criteria for significance were at least as stringent as those used in previous studies, and thus we believe that this strategy ensured that our results were comparable, or perhaps even more robust.

With the experimental design and behavioral results, the observed RT-predictive ongoing activity strongly suggests that intrinsic brain dynamics in a wide range of TCNs can be a primary source of performance fluctuation in situations where the demand for executive control occurs unpredictably. Nevertheless, we cannot totally exclude possible influences that task-driven processes may have had on performance-predictive ongoing activity. It has been observed that intrinsic and task-related neural activity are not always superimposed in an additive way, but sometimes interact with each other to generate the observed signals [14], [15], [73]. Furthermore, many studies conducted over a long time scale have shown that the spatial and temporal characteristics of intrinsic brain activity are modulated by the current context or preceding experiences [2], [74]–[79]. Taken together, our results should be interpreted from a wider perspective regarding mechanisms for processing information in neural systems, and seen as further evidence that active information processing is shaped by the intrinsic activity interacting with task demands, a concept shared by theoretical frameworks such as the Bayesian inference engine [80] and the free energy principle [11].

## Conclusions

In summary, this study explored the possibility that ongoing, intrinsic brain dynamics, revealed as fluctuating activity in distributed executive-control-related TCNs, can predict trial-to-trial fluctuations in executive control performance. We conducted an fMRI study using a version of the color–word Stroop task with higher cognitive load, with the aim of achieving increased sensitivity to fluctuations in executive control, and explored the relationships between fluctuations in ongoing pre-trial activity of TCNs and the task response time (RT). The results revealed distributed TCNs, especially prominent in the cingulo-opercular network, for which fluctuations in activity several seconds before the onset of the trials predicted RT fluctuations of the subsequent trials. This suggests that fluctuations in intrinsic activity lead to fluctuations in executive control performance. Intrinsic activity in the reported TCNs, reflecting their interactions with task demands, shapes “cognitive readiness”, which plays an active role even in situations where information for anticipatory attention control is unavailable. Apart from acquiring a theoretical understanding of the effects of intrinsic neural dynamics on executive control functions, the results of these and future studies may also have a great impact from a practical point of view. Since the demand for executive control can be quite sporadic and unpredictable, yet acute in daily life situations (e.g., driving a car), decoding such a state of cognitive readiness could help to develop technologies for improving safety or enhancing the efficiency of various forms of human intellectual activity.

## Supporting Information

Figure S1 **Description of all temporally coherent networks (TCNs), divided into seven network groups.** Spatial maps, BOLD activity around the trial onsets, and the relationship between their activity and response time (RT) for the TCNs in the (A) cingulo-opercular network (CON); (B) fronto-parietal network (FPN); (C) default mode network (DMN); (D) visual network; (E) auditory network; (F) sensorimotor network; (G) subcortical network. The format for composite visualization of each TCN is the same as in Figure 4. TCNs in solid boxes are those for which pre-trial activity (−6.0 s ≤ *t* ≤ 0.0 s relative to the task onset) predicted the RTs; dashed boxes indicate TCNs of which the activity at 0.0 < t ≤ +3.0 s from the trial onsets significantly explained RT variability. Cyan boxes indicate negative RT predictivity (higher early activity predicted faster response); orange boxes indicate positive RT predictivity (higher early activity predicted slower response).
(PPTX)

Figure S2 **Functional network connectivity (FNC) between all temporally coherent networks (TCNs) for resting session time courses (A) and for task session time courses without regressing out the average task-evoked responses (B).** The procedure for calculating and visualizing the FNC is the same as in Figure 3.
(PPTX)
